# The microbe-secreted isopeptide poly-γ-glutamic acid induces stress tolerance in *Brassica napus* L. seedlings by activating crosstalk between H_2_O_2_ and Ca^2+^

**DOI:** 10.1038/srep41618

**Published:** 2017-02-13

**Authors:** Peng Lei, Xiao Pang, Xiaohai Feng, Sha Li, Bo Chi, Rui Wang, Zongqi Xu, Hong Xu

**Affiliations:** 1 College of Food Science and Light Industry, Nanjing Tech University, Nanjing 211816, PR China; 2 Jiangsu National Synergetic Innovation Center for Advanced Materials, Nanjing Tech University, Nanjing 211816, PR China

## Abstract

Poly-γ-glutamic acid (γ-PGA) is a microbe-secreted isopeptide that has been shown to promote growth and enhance stress tolerance in crops. However, its site of action and downstream signaling pathways are still unknown. In this study, we investigated γ-PGA-induced tolerance to salt and cold stresses in *Brassica napus* L. seedlings. Fluorescent labeling of γ-PGA was used to locate the site of its activity in root protoplasts. The relationship between γ-PGA-induced stress tolerance and two signal molecules, H_2_O_2_ and Ca^2+^, as well as the γ-PGA-elicited signaling pathway at the whole plant level, were explored. Fluorescent labeling showed that γ-PGA did not enter the cytoplasm but instead attached to the surface of root protoplasm. Here, it triggered a burst of H_2_O_2_ in roots by enhancing the transcription of *Rboh*D and *Rboh*F, and the elicited H_2_O_2_ further activated an influx of Ca^2+^ into root cells. Ca^2+^ signaling was transmitted via the stem from roots to leaves, where it elicited a fresh burst of H_2_O_2_, thus promoting plant growth and enhancing stress tolerance. On the basis of these observation, we propose that γ-PGA mediates stress tolerance in *Brassica napus* seedlings by activating an H_2_O_2_ burst and subsequent crosstalk between H_2_O_2_ and Ca^2+^ signaling.

Abiotic stresses such as salinity, drought, and extreme temperatures, have a significant impact on plant growth and crop yield worldwide. It has been estimated that worldwide approximately 70% of yield reduction is the direct result of abiotic stresses^1^. Different methodologies have accordingly been employed with the aim of enhancing multiple stress tolerance, which mainly include plant genetic modification and exogenous application of plant growth regulators (PGRs), such as ethylene, brassinolide, and abscisic acid^2^,^3^,^4^. In many countries, however, plant genetic modification remains a very controversial issue because of potential food safety concerns^5^,^6^. Conversely, exogenous application of PGRs is a widely accepted measure in many countries^7^. Although most of these PGRs are hormonal chemicals that are considered to have low toxicity, research has shown that hormonal PGRs in waterbodies may endanger aquatic ecosystems^8^. Moreover, improper use of these PGRs in food crops could potentially affect mammalian reproductive fertility or increase estrogenic activity^9^. Therefore, many agronomists are endeavoring to develop more environmentally friendly PGRs that have no toxic effects on the environment or humans.

A number of studies have, nevertheless, reported a variety of biodegradable, non-toxic polymers that promote growth and enhance stress tolerance in plants. These include chitosan, polylactic acid, and polyaspartic acid^10^,^11^,^12^,^13^. Poly-γ-glutamic acid (γ-PGA), a bacterially secreted non-ribosomal peptide composed of amide-linked d- and l- glutamic acid monomers, is structurally similar to polyaspartic acid^14^—its monomers differing from those of polyaspartic acid by only a single additional methylene. On the basis of this similarity, we investigated the effects of γ-PGA on growth in wheat, and found that it produced increases in both yield (>7%) and nitrogen-recovery efficiency (>11%)^15^. Furthermore, both the quantity and the diversity of microorganisms found in associated soil cultures were increased following treatment with γ-PGA^16^. Recent results have further shown that γ-PGA enhances the tolerance of plants to certain abiotic stresses, such as cold and salt^17^,^18^. These findings, in conjunction with other known properties of γ-PGA (including chelating ability, hygroscopicity, and biodegradability) suggest that γ-PGA is a promising greener plant growth regulator.

However, whilst γ-PGA has been shown to alter the expression of certain genes, enhance the activity of antioxidant enzymes, and promote accumulation of osmoregulatory substances, the mechanism whereby the stress tolerance of plants is enhanced in response to γ-PGA treatment has yet to be fully elucidated^17^,^18^. Moreover, it is not known whether γ-PGA is taken up by plant cells in the form of a polypeptide biomacromolecule. Considering that another biomacromolecule, chitosan, plays positive roles in enhancing stress tolerance by activating associated signal molecules^19^, we assume that γ-PGA is most likely to promote crop growth by eliciting signaling activity.

Among numerous signaling molecules, H_2_O_2_, which has the longest lasting activity of all reactive oxygen species (ROS), plays a key role in early cellular signal transduction pathways, being involved in the regulation of growth, development, responses to environmental stimuli, and cell death^20^,^21^,^22^. In a previous study, we demonstrated that certain antioxidant enzymes can be activated by γ-PGA, namely, catalase (CAT), superoxide dismutase (SOD), and peroxidase (POD)^17^,^18^. These enzymes can be induced by H_2_O_2_ and form part of the H_2_O_2_ clearing system^23^, suggesting a possible link between the mechanism of the γ-PGA response and H_2_O_2_ signaling. In addition, Ca^2+^ signaling has also been implicated in the response to γ-PGA in plants according to our previous study^18^. Investigation of both the role of H_2_O_2_ and any possible link between H_2_O_2_ and Ca^2+^ signaling is therefore crucial in order to further elucidate the mechanism of the plant γ-PGA response.

In this study, we investigated the γ-PGA-induced tolerance to salt and cold stresses in rape (*Brassica napus* L.) seedlings. Fluorescent labeling of γ-PGA was used to locate the site of its activity in root protoplast. The relationship between γ-PGA-induced stress tolerance and two signal molecules, H_2_O_2_ and Ca^2+^, as well as the γ-PGA-elicited signal interaction of H_2_O_2_ and Ca^2+^ in the whole plant were explored.

## Results

### Effects of γ-PGA on growth parameters and physiological states in rape seedlings under salt and cold stresses

**Figure 1:**
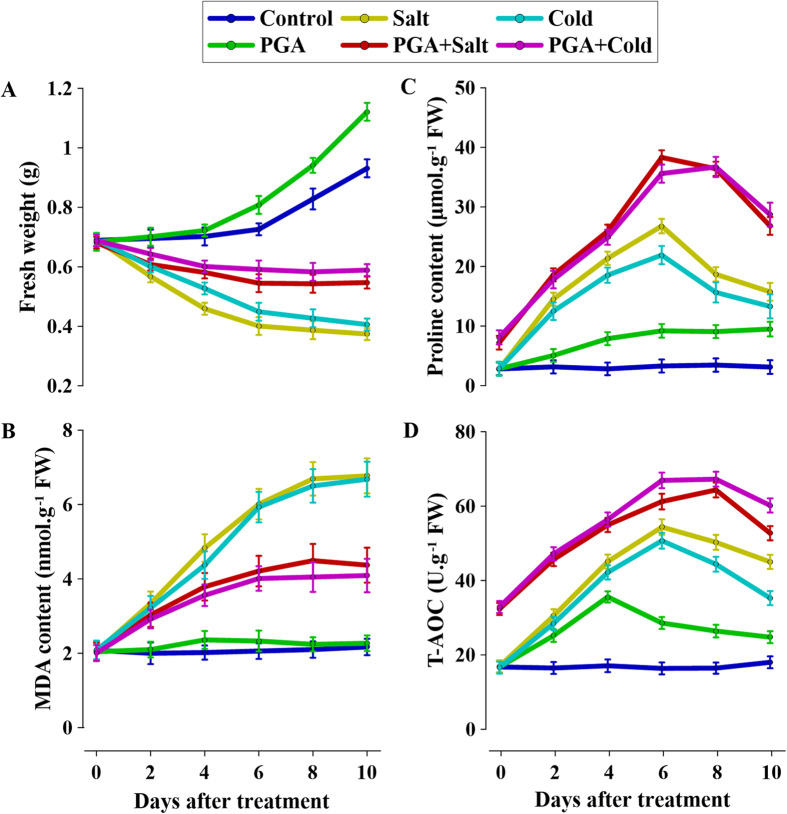
Effects of poly-γ-glutamic acid (γ-PGA) on the growth and physiological parameters of *Brassica napus* seedlings subjected to salt and cold stresses. (**A**) fresh weight; (**B**) malonaldehyde (MDA) content; (**C**) proline content; (**D**) total anti-oxidative capacity (T-AOC).

Salt and cold stresses significantly inhibited the growth of rape seedlings, which was manifested as a decrease in fresh weight (Fig. 1A). However, pre-treatment with γ-PGA reduced the decrease in fresh weight under stress conditions. In addition, changes in physiological indices such as MDA content, proline content, and T-AOC were examined under stress conditions. MDA is the main product of membrane lipid peroxidation caused by excess ROS in stressed plants, the content of which can reflect the degree of membrane damage caused by abiotic stresses. Plants generally enhance stress tolerance by accumulating proline and improving T-AOC. In this study, stresses increased the MDA contents in leaves, although this increase was reduced to some extent with pre-treatment of γ-PGA (Fig. 1B). Proline content and T-AOC in leaves were significantly increased in seedlings subjected to salt and cold stresses (Fig. 1C,D). However, the levels of these two physiological indices were much higher in stressed seedlings following pre-treatment with γ-PGA. Moreover, addition of γ-PGA induced increases in T-AOC and proline content in leaves to some extent even in non-stressed seedlings.

### Fluorescence tracing of γ-PGA in root cells

**Figure 2:**
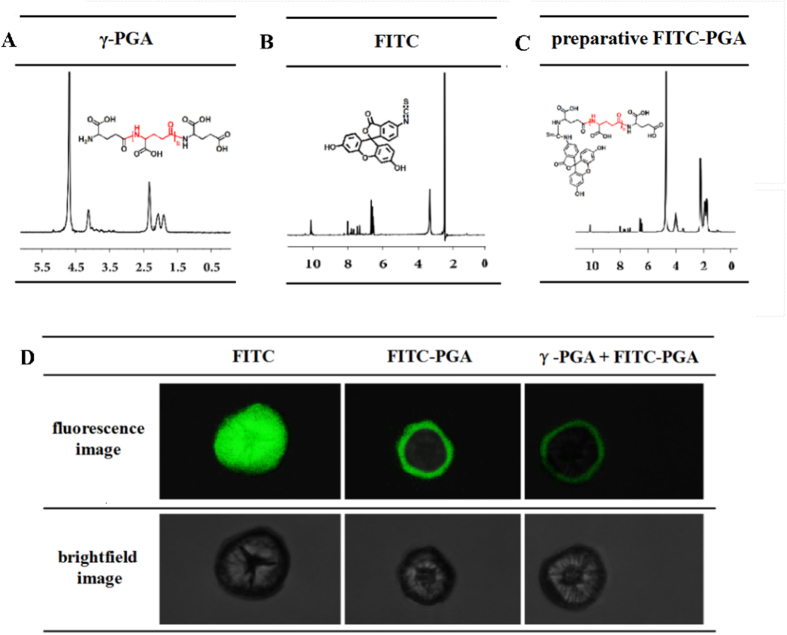
The fluorescence tracing of poly-γ-glutamic acid (γ-PGA) in *Brassica napus* root protoplasts. (**A**) The ^1^H-NMR spectra of γ-PGA; (**B**) the ^1^H-NMR spectra of fluorescein isothiocynate (FITC); (**C**) the ^1^H-NMR spectra of preparative FITC-PGA; (**D**) laser scanning confocal microscope (LSCM) scanning images of root protoplasts after treatment with FITC, FITC-PGA, or γ-PGA plus FITC-PGA, respectively.

As shown in Fig. 2, the structures γ-PGA, fluorescein isothiocyanate (FITC: a fluorescent dye), and the preparative FITC labeled γ-PGA (FITC-PGA) were characterized using 1 H NMR. The results showed that the preparative FITC-PGA had characteristic peaks of both γ-PGA and FITC, which indicated that FITC-PGA was successfully prepared (Fig. 2C). Fluorescence tracing revealed that FITC could enter root protoplasts but that FITC-PGA was unable to cross the plasma membrane (Fig. 2D). However, when an equal amount of γ-PGA was pre-added before the addition of FITC-PGA, the amount of FITC-PGA that attached to the surface of the plasma membrane was markedly decreased and the fluorescence intensity became weaker.

### Effects of γ-PGA on H_2_O_2_ content in rape seedling roots

**Figure 3:**
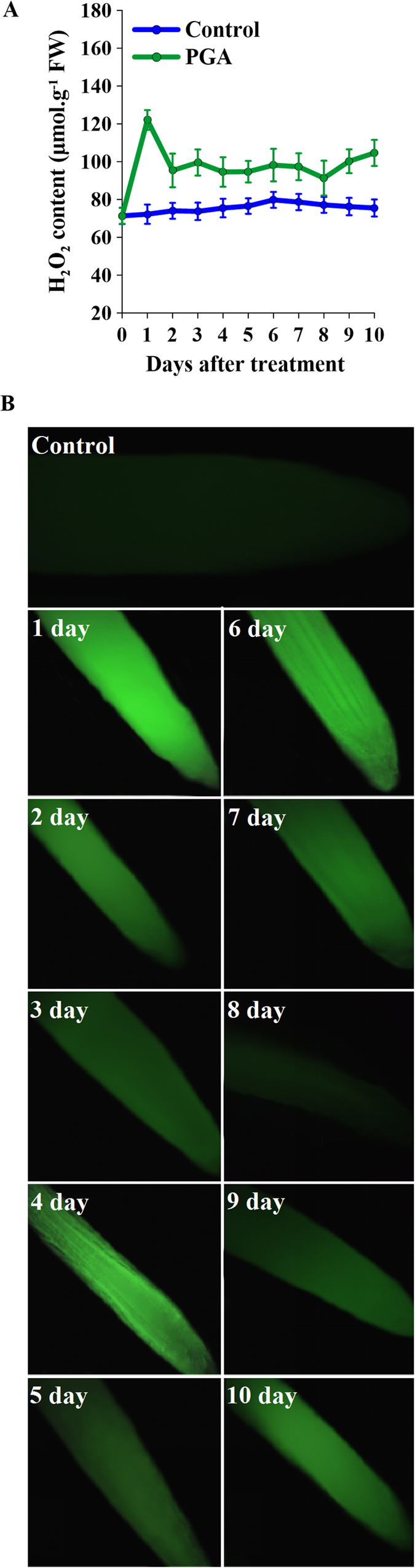
Changes in H_2_O_2_ content in *Brassica napus* seedling roots after treatment with poly-γ-glutamic acid (γ-PGA). (**A**) Changes in the H_2_O_2_ content of roots following application of γ-PGA. (**B**) Dichloro-dihydro-fluorescein diacetate (DCFH-DA) fluorescence images of rape seedling roots after treatment with γ-PGA.

H_2_O_2_ levels in roots were significantly increased after the application of γ-PGA (Fig. 3A). At day 1 post-treatment, H_2_O_2_ increased rapidly to a peak, and then gradually decreased over the subsequent days, although it continued to remain at a significantly higher level than in the control. The staining patterns of dichloro-dihydro-fluorescein diacetate (DCFH-DA: a fluorescent dye used for detecting H_2_O_2_) in the root tips were consistent with these measured results (Fig. 3B). Following treatment with γ-PGA, all of the tested root tips showed brighter fluorescence than the control, with the greatest intensity being observed in the tip at 1 day post γ-PGA treatment. In the control group, all root tips sampled on different days showed considerably weaker fluorescence and differences among the different samples were non-significant. Accordingly, only one image of these control roots has been shown in Fig. 3B.

### The relationship between H_2_O_2_ elicited by γ-PGA and the stress tolerance of rape seedlings

**Figure 4:**
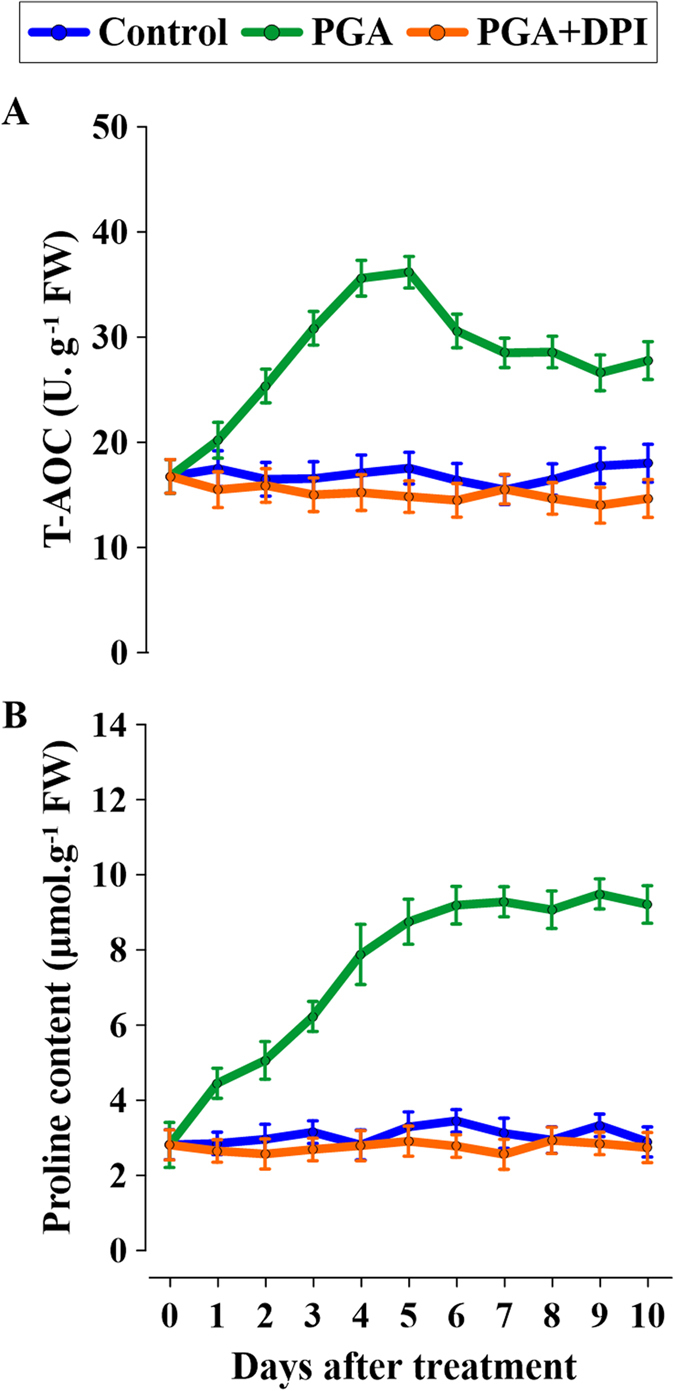
Effects of H_2_O_2_ elicited by poly-γ-glutamic acid (γ-PGA) on (**A**) total anti-oxidative capacity (T-AOC) and (**B**) proline content.

In this study, we used the T-AOC and proline content as indicators of stress tolerance in rape seedlings. As shown in Fig. 4A, compared with the control, γ-PGA significantly increased the T-AOC of rape seedling leaves. However, diphenylene iodonium (DPI: an inhibitor of H_2_O_2_ production) almost completely inhibited this increase in T-AOC caused by γ-PGA. Similarly, the proline content in leaves was also significantly increased by γ-PGA, and this increase was also inhibited by DPI (Fig. 4B).

### Tissue localization of H_2_O_2_

**Figure 5:**
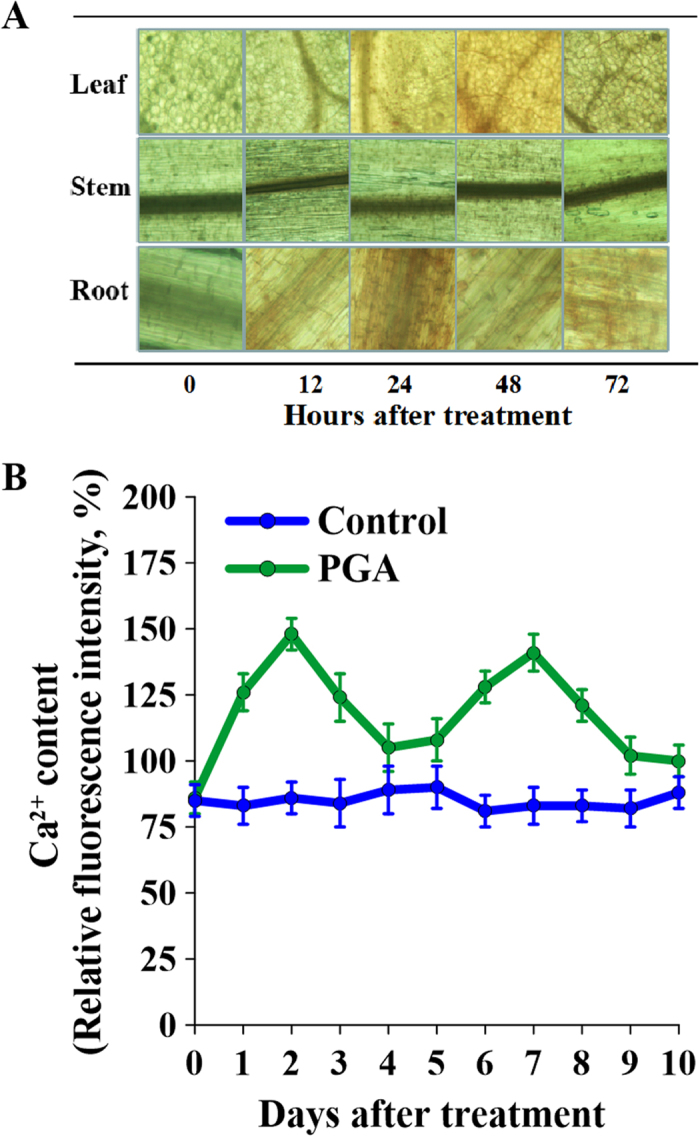
The tissue localization of H_2_O_2_ in the whole plant of *Brassica napus* and changes in Ca^2+^ content in root cytoplasm after treatment with poly-γ-glutamic acid (γ-PGA). (**A**) Tissue localization of H_2_O_2_ detected with 3,3′-diaminobenzidine (DAB) staining; the deeper the brown coloration, the higher the H_2_O_2_ content. (**B**) Ca^2+^ curves in roots treated with and without γ-PGA.

In order to investigate whether the localized H_2_O_2_ signaling elicited by γ-PGA in the roots was transmitted to the entire plant, we performed 3,3′-diaminobenzidine (DAB) staining. The higher the concentration of H_2_O_2_ in a tissue, the more intense was the brown staining observed (Fig. 5A). H_2_O_2_ content was seen to increase in response to γ-PGA in both roots and leaves. In the roots, the H_2_O_2_ content was highest at 24 h post-treatment, which was consistent with DCFH-DA staining results presented above, whereas the H_2_O_2_ content in the leaves peaked at 48 h. However, we did not observe any difference in staining in the stems, suggesting that H_2_O_2_ release in response to γ-PGA was transmitted from roots to leaves without flowing through stems.

### Changes in Ca^2+^ content induced by γ-PGA

Cytoplasmic Ca^2+^ content was also significantly increased in the roots after application of γ-PGA (Fig. 5B). However, in contrast to the steady pattern observed for H_2_O_2_, Ca^2+^ showed a more volatile pattern of temporal flux. Cytoplasmic Ca^2+^ reached a peak at day 2 post-treatment, and then decreased to levels similar to those in the control by day 4. This pattern was then repeated, peaking again at day 6 and then decreasing until day 10.

### γ-PGA-induced changes in the fluxes of H_2_O_2_ and Ca^2+^ signaling

**Figure 6:**
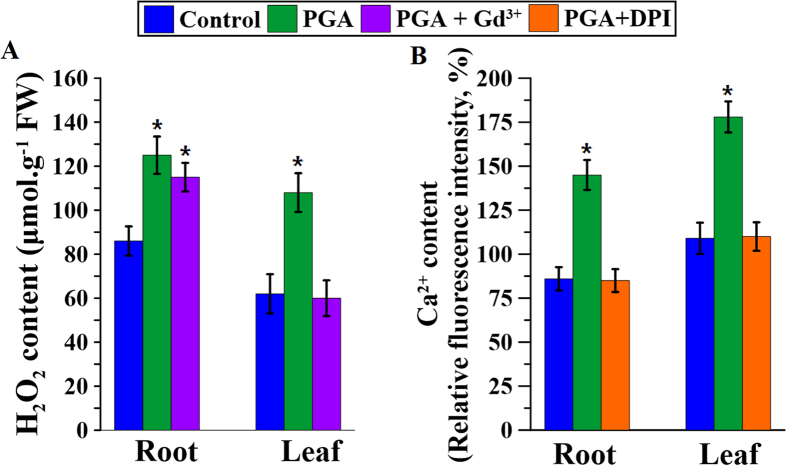
H_2_O_2_ and Ca^2+^ contents in roots and leaves of Brassica napus seedlings after treatment with poly-γ-glutamic acid (γ-PGA) and corresponding signal blockers. (**A**) H_2_O_2_ contents; (**B**) Ca^2+^ contents. *Indicates that there was a significant difference between treatment and control groups (*p* < 0.05).

In order to investigate the relationship between γ-PGA-activated H_2_O_2_ and Ca^2+^ signaling, the levels of these two signaling molecules in the roots and leaves were measured in the presence of different blockers. As shown in Fig. 6A, Gd^3+^ (a blocker of Ca^2+^ signaling) inhibited the increase in H_2_O_2_ promoted by γ-PGA in leaves, but had no effect in roots, whereas DPI inhibited the γ-PGA-induced Ca^2+^ increase in both roots and leaves (Fig. 6B).

**Figure 7:**
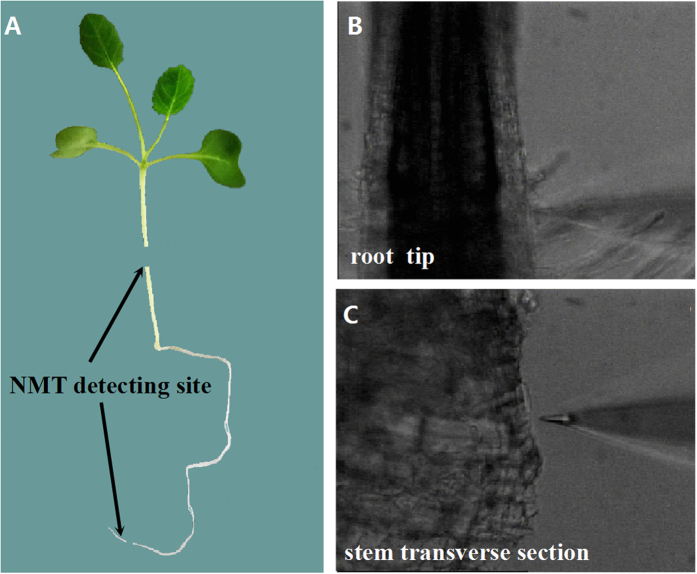
Use of the non-invasive micro-test technique (NMT) to detect fluxes of H_2_O_2_ and Ca^2+^ in Brassica napus seedlings. (**A**) Detection sites in the whole plant; (**B**) microscopic image of detection in the root tip; (**C**) microscopic image of detection in a stem transverse section.

**Figure 8:**
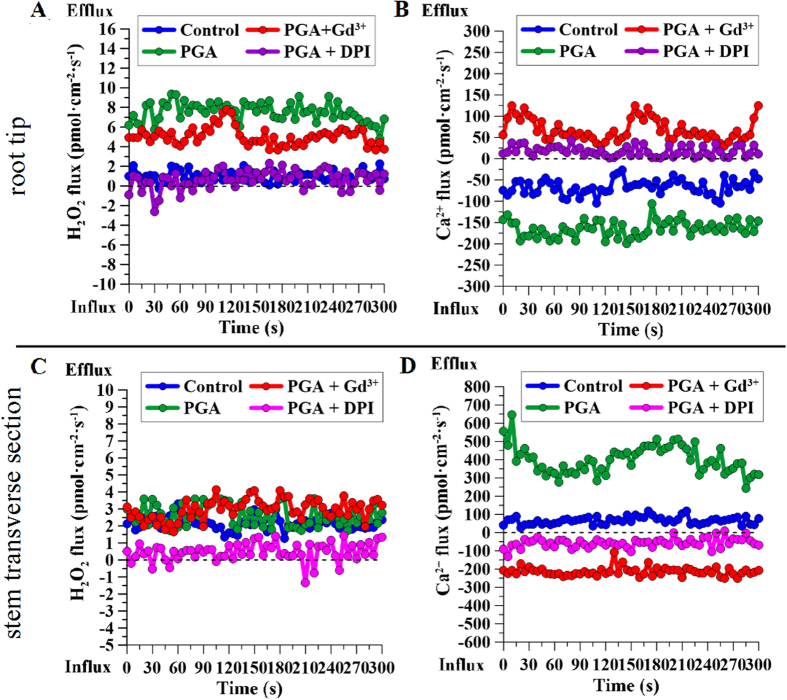
Fluxes of Ca^2+^ and H_2_O_2_ in root tips and stem transverse sections of Brassica napus seedlings after treatment with poly-γ-glutamic acid (γ-PGA), and changes in Ca^2+^ and H_2_O_2_ in the presence of different signal blockers, detected using the non-invasive micro-test technique (NMT). (**A**) H_2_O_2_ fluxes in the root tip; (**B**) Ca^2+^ fluxes in the root tip; (**C**) H_2_O_2_ fluxes in a stem transverse section; (**D**) Ca^2+^ fluxes in a stem transverse section.

To further elucidate the relationship between H_2_O_2_ and Ca^2+^ signaling, the non-invasive micro-test technique (NMT) was used to detect the fluxes in H_2_O_2_ and Ca^2+^ in different parts of rape seedlings (Fig. 7). NMT, which was developed based on a “vibrating probe^24^,” non-invasively shows how fast Ca^2+^ and/or H_2_O_2_ enter or leave living samples (Fig. 7B,C). In the root tips, γ-PGA enhanced the H_2_O_2_ efflux from root cells, but this enhancement was inhibited by pre-addition of DPI (Fig. 8A). Pre-addition of Gd^3+^ had no significant effect on the H_2_O_2_ efflux elicited by γ-PGA. γ-PGA also enhanced the Ca^2+^ influx and this enhancement was inhibited by pre-addition of both Gd^3+^ and DPI (Fig. 8B). In stem transverse sections (Fig. 8C,D), γ-PGA stimulation only enhanced the Ca^2+^ efflux and had no significant effect on the H_2_O_2_ flux. However, the Ca^2+^ efflux activated by γ-PGA in the stem was nonetheless inhibited by pre-addition of DPI.

### The effects of γ-PGA on H_2_O_2_-producing NADPH oxidases

**Figure 9:**
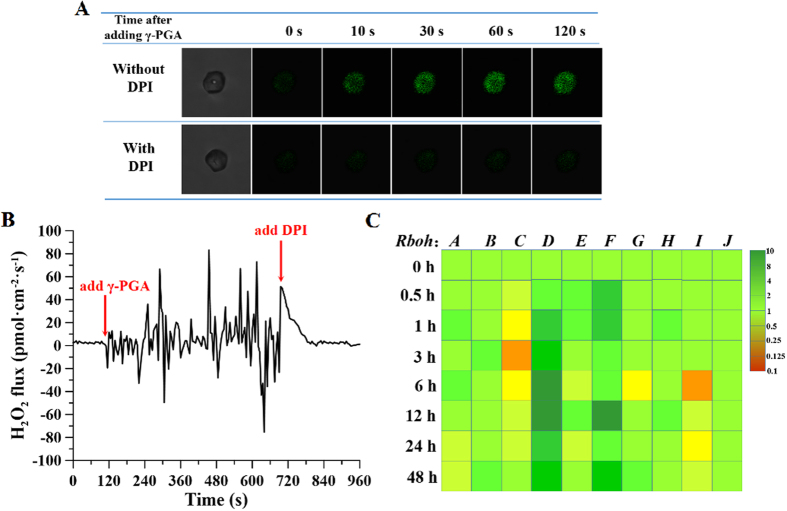
The relationship between poly-γ-glutamic acid (γ-PGA)-activated H_2_O_2_ and NADPH oxidases. (**A**) Fluorescence staining with dichloro-dihydro-fluorescein diacetate (DCFH-DA) in *Brassica napus* root protoplasts after treatment with or without diphenylene iodonium (DPI) in the presence of γ-PGA; (**B**) instantaneous real-time fluxes of H_2_O_2_ following addition of γ-PGA or DPI; (**C**) relative transcription levels of *Rboh* genes in roots after treatment with γ-PGA.

To elucidate the mechanism by which γ-PGA influences H_2_O_2_ production in the roots, the transient effects of γ-PGA on H_2_O_2_ levels in root protoplasts were investigated. DCFH-DA staining showed that the H_2_O_2_ content in protoplasts increased rapidly within 30 s after the application of γ-PGA. However, pre-treatment of protoplasts with DPI, which inhibits NADPH oxidases, prior to γ-PGA treatment led to few significant changes in H_2_O_2_ content (Fig. 9A).

Transient effects of γ-PGA on the H_2_O_2_ flux in root protoplasts were also detected using NMT. As shown in Fig. 9B, the H_2_O_2_ flux was initially at stable levels in the base solution. On addition of γ-PGA, marked fluxes in H_2_O_2_ levels began to be observed, which were subsequently inhibited by the addition of DPI to the base solution. Figure 9C shows the effects of γ-PGA treatment on the relative transcription levels of 10 *Rboh* genes (NADPH oxidase genes). γ-PGA treatment had no significant effect on the majority of *Rboh* genes (*Rboh A, B, E, G, H, J*), although slight inhibition of *Rboh C* and *Rboh I* transcription was observed. In contrast, the relative transcription levels of both *Rboh D* and *Rboh F* were significantly up-regulated in response to γ-PGA treatment.

## Discussion

In this study, γ-PGA significantly reduced the fresh weight decrease and the MDA increase caused by salt and cold stresses (Fig. 1), which indicated that γ-PGA can contribute to the enhancement of stress tolerance in rape seedlings. These results are consistent with those of our previous studies^17^,^18^. In addition, we found that γ-PGA increased proline content and T-AOC, even in the absence of stress, suggesting that γ-PGA itself could lead to physiological changes in rape seedlings. The levels of these two physiological indices also increased under stress conditions, which indicated they are related to stress tolerance in plants. In this regard, variation between species and genotypes in tolerance to various environmental stresses has been linked to leaf antioxidant capacity and proline content^1^,^25^, and thus these indices can be considered potentially useful phenotypic markers of stress tolerance. We therefore deduced that γ-PGA enhanced the stress tolerance of rape seedlings by promoting a stress-resistant status.

In order to further study the mechanism whereby γ-PGA induces stress tolerance in rape seedlings, we initially needed to know whether this macromolecule can be absorbed by plants. γ-PGA is a microbially secreted anionic polypeptide with a molecular weight greater than 200 kDa^26^. The γ-PGA used in the present study had a molecular weight of 2 kDa, which was previously shown to be the most effective in stimulating plant growth^27^. Uptake of these exogenous macromolecules is typically limited in plant roots. Consistent with this, the fluorescent-labeling results obtained in this study showed that γ-PGA is unable to enter the cytoplasm, but instead attaches to the membrane surface of root cell protoplasts (Fig. 2D). Although there have been previous reports on the uptake of certain macromolecules, these have been limited to polycationic compounds, such as poly-l-lysine, lysozyme, and β-lactoglobulin^28^. γ-PGA carries a negative surface charge, which may explain its inability to enter the cytoplasm. Furthermore, pre-treatment with non-labeled γ-PGA significantly reduced the localization of FITC-PGA on the root protoplast membrane, suggesting that there may be specific binding sites for γ-PGA on the root cell membrane. There are other examples of macromolecules that can influence plant growth despite lack of cellular uptake. These include oligosaccharides that can both induce phytoalexin synthesis and promote plant growth, such as oligoglucoside, chitin oligosaccharide, and glycopeptides, all of which exert their growth effects by cell membrane attachment^29^,^30^. These macromolecules regulate plant defense capability through stimulating cells to produce defense signals, and specific binding proteins within the plant cell membrane have been identified for some of these^31^. It is therefore likely that γ-PGA influences plant growth by stimulating a signaling cascade within the cell via membrane binding.

As previously discussed, H_2_O_2_ is an important signal molecule in plants. Plants produce H_2_O_2_ in response to stresses such as climatic change, pathogen invasion, and environmental chemical agents in order to promote growth, and this is considered to be an early stress response signal^20^,^32^. There have been reports of other polymers that regulate plant growth by eliciting a burst of H_2_O_2_^33^,^34^. The inability of γ-PGA to enter root cell cytoplasm suggests that its role may be mediated via H_2_O_2_ signaling. The present study demonstrated that γ-PGA triggers a burst of H_2_O_2_ in rape seedling roots and that this increase in H_2_O_2_ was sustained for over 10 days, indicating a long-term effect on rape seedlings. However, we detected no increase in MDA produced by membrane oxidation concomitant with an increase in H_2_O_2_ after application of γ-PGA (Fig. 1B), which indicates that the elevated level of H_2_O_2_ was not harmful to plant cells but instead acts as a signal. In order to confirm that the γ-PGA-enhanced stress tolerance in plants was related to the H_2_O_2_ burst, T-AOC and proline content were measured as markers of stress tolerance (Fig. 4). We observed that both the T-AOC and proline content in leaves were increased by γ-PGA, indicating an enhancement of stress tolerance in rape seedlings. However, this enhancement was completely inhibited by DPI, a blocker of H_2_O_2_ production. Therefore, we deduced that γ-PGA-enhanced stress tolerance in rape seedlings is related to H_2_O_2_ signaling.

Interestingly, we found that, although γ-PGA treatment led to an increase in H_2_O_2_ contents in both the roots and the leaves, no significant changes were observed in stems, suggesting that the H_2_O_2_ signaling jumped between tissues (Fig. 5A). It has previously been shown that a H_2_O_2_ wave triggered by different stimuli can be blocked by the local application of CAT at distances of up to 5–8 cm from the signal initiation site^35^. Since plants have a high capacity to scavenge H_2_O_2_, long-distance H_2_O_2_ signaling is difficult to achieve without a continuous production of H_2_O_2_ in individual cells along the path of H_2_O_2_ signaling^35^,^36^. γ-PGA, however, only acts on the surface of roots, producing local stimulation. It is therefore possible that the H_2_O_2_ wave elicited by γ-PGA in the roots was cleared by the antioxidant system before spreading to the stem. Furthermore, H_2_O_2_ generally functions as an initial signal to activate the further downstream cellular signaling networks^37^. Therefore, there may be other signals mediating the root to leaf transmission of H_2_O_2_.

In previous studies, we have shown that calcium signaling is implicated in the γ-PGA-mediated promotion of nitrogen metabolism in Chinese cabbage and enhancement of cold resistance in rape seedlings^18^,^27^. However, the causes of the observed fluxes in Ca^2+^ were not investigated in these previous studies. The current study demonstrated that in addition to promoting fluxes in H_2_O_2_, γ-PGA also elicited intracellular fluctuations of Ca^2+^ in roots, thereby indicating a possible relationship between H_2_O_2_ and Ca^2+^ signaling. At least two different functional processes link Ca^2+^ and H_2_O_2_ signaling in plant cells: Ca^2+^ -induced H_2_O_2_-production and H_2_O_2_-induced Ca^2+^ -release. In the case of the former, Ca^2+^ can activate NADPH oxidases to produce H_2_O_2_. Moreover, although Ca^2+^ -based regulation is required for the activation of NADPH oxidases, other regulatory processes are likely to exist^38^. In the case of the second possibility, H_2_O_2_ can directly activate a membrane Ca^2+^ channel or pump to increase the uptake of Ca^2+^ 39,40. In the present study, Ca^2+^ signaling in both the roots and leaves was regulated by γ-PGA-elicited H_2_O_2_, although H_2_O_2_ signaling was regulated by γ-PGA-activated Ca^2+^ only in leaves (Fig. 6), indicating that H_2_O_2_ is an the upstream signal in rape seedling responding to γ-PGA and that Ca^2+^ is an intermediate signaling molecule.

The results of NMT detection in the root tip and stem transverse sections further confirmed this supposition. In the root tip, γ-PGA treatment enhanced the H_2_O_2_ efflux and Ca^2+^ influx, which was consistent with changes in their contents after treatment with γ-PGA. Furthermore, the influx of Ca^2+^ was regulated by DPI, whereas the H_2_O_2_ efflux was not regulated by Gd^3+^, suggesting that Ca^2+^ signaling operates downstream of H_2_O_2_ signaling in roots. In this regard, it has been reported that H_2_O_2_ activates hyperpolarization-activated Ca^2+^ -permeable channels in plant cells^41^,^42^. Therefore, we conjecture that H_2_O_2_ signaling was initially elicited by γ-PGA in roots and subsequently activated Ca^2+^ signaling by activating Ca^2+^ -permeable channels. In stem transverse sections, γ-PGA stimulation resulted in an increased Ca^2+^ efflux, suggesting that the Ca^2+^ signal was transmitted to leaves. However, γ-PGA had no direct effect on the flux of H_2_O_2_ in stems, suggesting that the Ca^2+^ flux from the stem re-triggered an H_2_O_2_ burst in leaves. The Ca^2+^ efflux from intracellular to extracellular sites typically depends on Ca^2+^ -ATPase, the activity of which can be enhanced by Ca^2+^ 43. Application of the H_2_O_2_ signaling inhibitor DPI also inhibited the efflux of Ca^2+^ in the stem, indicating that Ca^2+^ signaling in the stem is regulated by H_2_O_2_ signaling in the roots. Therefore, it can be concluded that the signaling path elicited by γ-PGA in roots shows the following sequence: “γ-PGA → H_2_O_2_ → Ca^2+^”. Ca^2+^ is then transported from roots to leaves via the stem, and in leaves it activates H_2_O_2_ “Ca^2+  → ^H_2_O_2_”. The activated H_2_O_2_ in leaves can promote plant growth and regulate plants to respond to environmental stress by adjusting physiological changes, such as increasing T-AOC and proline content.

Having demonstrated that γ-PGA enhances stress tolerance in plants by triggering an H_2_O_2_ burst, we went on to explore the transient effect of γ-PGA on the H_2_O_2_ burst in root protoplasts in order to determine the mechanism by which γ-PGA triggers the H_2_O_2_ burst. Higher plants have a most complex H_2_O_2_ metabolism under optimal environmental conditions, which is in part a consequence of the photosynthesis and photorespiration processes. H_2_O_2_ is a typical side product of many physiological pathways that are present in all cell compartments, including chloroplasts, mitochondria, plasma membrane, and peroxisomes^44^. Considering that γ-PGA was not transported into the cytoplasm, it is most likely to trigger an H_2_O_2_ burst by interacting with the plasma membrane. NADPH-dependent oxidases (NADPH oxidases) associated with plasma membranes have recently been identified as a source of H_2_O_2_ in the cellular oxidative bursts that are typical responses to environmental stimuli^45^. Inhibitors of NADPH oxidases, such as DPI, have been shown to block or severely reduce H_2_O_2_ production in response to biotic and abiotic stresses^46^. As anticipated, the addition of γ-PGA elicited an H_2_O_2_ increase in root protoplasts within 30 s, and this immediate effect did not occur following pre-addition of DPI. In addition, NMT detection showed that γ-PGA indeed caused an instant and strong H_2_O_2_ flux in root protoplasts and that this fluctuation was inhibited by the addition of DPI. This suggests that the burst of H_2_O_2_ elicited by γ-PGA was associated with NADPH oxidases. In the present study, we determined the transcription levels of 10 *Rboh* genes, which correspond to the *Rboh* genes of *Arabidopsis thaliana*, at sequences from A to J in rape seedling roots. *Rboh D* and *Rboh F* transcription levels were found to significantly increase in response to γ-PGA. Although there are 10 *Rboh* genes in *B. napus*, their relative expression levels are associated with different environmental stimuli, tissues, and cell types. Current studies of *Rboh* gene expression are focused primarily on the model plant, *Arabidopsis thaliana. AtRboh* D and *AtRboh* F have been shown to be involved in multiple environmental stress responses and play a key role in H_2_O_2_ production^47^. Taken together, these results indicate that γ-PGA may elicit H_2_O_2_ through the upregulation of RBOH D and RBOH F activities in roots.

**Figure 10:**
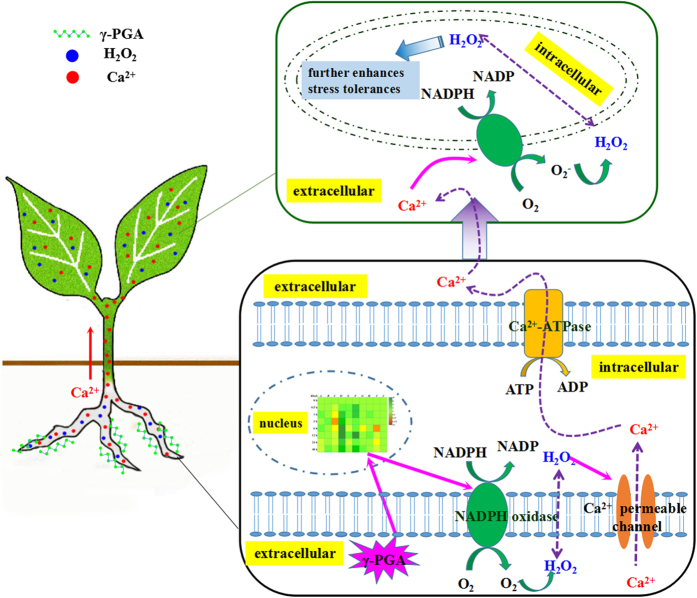
Proposed signaling pathways in crops elicited by treatment with poly-γ-glutamic acid (γ-PGA).

In conclusion, in the present study, we investigated the site of action of γ-PGA in rape seedling root cells, and identified possible downstream signaling pathways that regulate stress tolerance. On the basis of the results of this study, we propose the following signaling pathway in rape seedlings in response to γ-PGA (Fig. 10): γ-PGA does not enter the cytoplasm but instead attaches to the surface of root cell protoplasts; an H_2_O_2_ burst is elicited within the root cells by enhancing the transcription of *Rboh*D and *Rboh* F; H_2_O_2_ then activates Ca^2+^ permeability channels, leading to an influx of Ca^2+^ in root cells; Ca^2+^ signaling is transmitted from roots to leaves via the stem and a new H_2_O_2_ burst is elicited in the leaves, which contributes to plant growth and enhanced stress tolerance. Accordingly, γ-PGA mediates stress tolerance in *Brassica napus* seedlings by activating an H_2_O_2_ burst and subsequent cross-talk between H_2_O_2_ and Ca^2+^ signaling.

## Methods

### Plant materials and growth conditions

The rape variety used in this study was Suyou No. 1 (*Brassica napus* L., acquired from the Jiangsu Academy of Agricultural Sciences). Seeds were sterilized and then germinated in split-level germinating boxes, consisting of an upper tray with numerous pores in the base that are used for placing seeds, and a lower tray, used for loading culture solution. After the seeds germinate, their roots extend down to the lower culture solution via the pores in the upper tray. Growth conditions were controlled using a light-dark cycle of 16 h light at 25 °C followed by 8 h of darkness at 15 °C, and a constant relative humidity of 65%. As a culture medium, we used half-strength modified Hoagland Solution (HS), which has the following composition: 6 mM KNO_3_, 4 mM Ca(NO_3_)_2_, 2 mM NH_4_H_2_PO_4_, 1 mM MgSO_4_, 0.05 mM KCl, 0.025 mM H_3_BO_3_, 0.002 mM MnSO_4_, 0.002 mM ZnSO_4_, 0.0005 mM H_2_MoO_4_, 0.0005 mM CuSO_4_, and 0.0128 mM EDTA-Fe. The solution was changed every 2 days and the seedlings exhibiting consistent growth status after 4 weeks were used for the following seven experiments.

### Experiment 1

Rape seedlings were divided into six groups and cultured as follows: Group 1 (Control) with half-strength modified HS only; Group 2 (PGA) with half-strength modified HS plus γ-PGA (2 kDa, 20 mg L^−1^, the molecular weight and dosage of γ-PGA used in this study were the same unless specified); Group 3 (Salt) with half-strength modified HS plus 100 mM NaCl; Group 4 (PGA + Salt) with half-strength modified HS plus γ-PGA and 100 mM NaCl; Group 5 (Cold) with half-strength modified HS only; and Group 6 (PGA + Cold) with half-strength modified HS plus γ-PGA. With the exception of Group 5 and Group 6 seedlings, which were cultured at 4 °C, all the other groups were cultured at 25 °C. For Group 4 and Group 6, γ-PGA was pre-added 96 h before stress treatment (NaCl and cold). Rape seedlings were sampled on a daily basis for 10 days. The fresh weight of whole plants, the malonaldehyde (MDA) content, total anti-oxidative capacity (T-AOC), and proline content in the leaves were determined.

Determinations of MDA and proline contents were performed as described previously^18^. For T-AOC determination, rape leaves (0.5 g) were homogenized in 1.5 mL of precooled Tris-HCl buffer (pH 7.5, containing 5% sucrose and 0.1% mercaptoethanol). The homogenate was centrifuged at 10000 × *g* at 4 °C for 20 min. The supernatant was used to determine the T-AOC using the ferric reducing-antioxidant power assay and was detected at spectrophotometrically at 520 nm^48^.

### Experiment 2

Protoplasts were prepared from harvested rape seedling roots. The harvested protoplasts were then stained with 10 μM fluorescein isothiocynate (FITC: a fluorescent dye that fluoresces green light at 450–480 nm), 10 μM FITC-PGA (γ-PGA labeled with FITC at the N terminus), and 10 μM FITC-PGA after a pretreatment with 10 μM γ-PGA, respectively. All treatment times were of 20 min duration. After washing three times by centrifugation, the protoplasts were observed and photographed under a laser scanning confocal microscope (LSCM).

FITC-PGA was prepared according to the method of Park *et al*.^49^, with modifications. γ-PGA (2%) was mixed with 0.5 mmol FITC in 50 mL of phosphate-buffered saline (PBS) solution (pH 7.4). The mixture was incubated for 16 h in the dark at 4 °C. A 3-fold volume of 100% alcohol was then added to the reaction mixture, which was then centrifuged at 1000 × *g*. The supernatant was discarded and the pellet was re-washed with 100% alcohol. The centrifugation and washing steps were then repeated until no fluorescence could be detected in the supernatant using a fluorospectrometer. The resulting FITC-PGA pellet was dried using a vacuum freeze-dryer and characterized using nuclear magnetic resonance (NMR) spectroscopy.

Root protoplasts were prepared according to the method of Xu *et al*.^50^, with modifications. Roots were harvested and cut into 1-mm segments. The segments were plasmolyzed in a CPW solution (27.2 mg L^−1^ KH_2_PO_4_, 101.0 mg L^−1^ KNO_3_, 1480.0 mg L^−1^ CaCl_2_·2H_2_O, 246.0 mg L^−1^ MgSO_4_·7H_2_O, 0.025 mg L^−1^ CuSO_4_·5H_2_O, 0.16 mg L^−1^ KI, pH 5.6) with addition of 13% mannitol (CPW-13M) for 1 h and then incubated in an enzyme solution consisting of 2% (w/v) Rhozyme, 4% Meicelase, and 0.3% Macerozyme in CPW-13M. The enzyme-protoplast mixture was filtered through a cell strainer (64 μm) and the filtrate was centrifuged at 100 × *g* for 10 min. The pellet was then re-washed twice by centrifuging (100 × *g*, 5 min). The final pellet was subsequently suspended in CPW-20S solution (CPW with addition of 20% sucrose).

### Experiment 3

Rape seedlings were cultured in half-strength modified HS, in either the presence or absence of added γ-PGA. The culture solution was renewed each day. After continuous daily sampling for 10 days, the H_2_O_2_ content in roots and its tissue localization in the whole plant were determined. In addition, the cytoplasmic Ca^2+^ content in roots was also determined.

The quantitative determination of H_2_O_2_ was carried out according to the method described by Frew *et al*.^51^ Rape tissues (0.5 g) were extracted using 3 mL cold acetone and the resultant extracts were centrifuged at 5 000 × *g* at 4 °C for 10 min. One milliliter of the supernatant was then mixed with 0.1 mL 5% TiSO_4_ and 0.1 mL ammonia. After centrifugation at 3000 × *g* for 10 min, the pellet was resuspended in 4 mL 2 M H_2_SO_4_. H_2_O_2_ contents were determined spectrophotometrically at 415 nm. The qualitative detection of H_2_O_2_ was carried out in accordance with the fluorescent indicator method^52^. Briefly, root tips were cut into small fragments and placed into a PCR tube containing 500 μL dichloro-dihydro-fluorescein diacetate (DCFH-DA) staining solution (10 μM). The tube was then incubated at 37 °C for 10 min, after which the root tips were washed three times with purified water and observed under a fluorescence microscope. The tissue localization of H_2_O_2_ was determined using the 3,3′-diaminobenzidine (DAB) staining method. Briefly, whole seedlings were vacuumized for 30 min and then immersed in 1 mg mL^−1^ DAB solution (prepared according to the method of Thordal-Christensen *et al*.^53^) for 24 h in the dark. The stained seedlings were transferred to 95% alcohol at 70 °C for 10 min and then observed under a light microscope.

Measurement of cytoplasmic Ca^2+^ content was performed using a previously described method^18^. Root protoplasts were isolated as described above. The isolated root protoplasts were washed and incubated with Fluo-3/AMester at 4 °C for 1 h and then 25 °C for 1 h in the dark. The incubation solution contained 10 mM Fluo-3/AMester, 0.4 M mannitol, 20 mM KCl, and 5 mM MES (pH 5.7). Photographs were taken by scanning three times every 30 s using a laser scanning confocal microscope. After establishing a stable baseline, the fluorescence intensities of the photographs were measured using fluorescence microscopy.

### Experiment 4

Rape seedlings were divided into three groups and cultured as follows: Group 1 (Control) with half-strength modified HS only; Group 2 (PGA) with half-strength modified HS plus γ-PGA; Group 3 (PGA + DPI) with half-strength modified HS plus γ-PGA and 100 μM diphenylene iodonium (DPI: an H_2_O_2_ signaling blocker). DPI was added 24 h before the addition of γ-PGA. After continuous daily sampling for 10 days, the T-AOC and proline content in the leaves were determined.

### Experiment 5

Rape seedlings were divided into four groups and cultured as follows: Group 1 (Control) with half-strength modified HS only; Group 2 (PGA) with half-strength modified HS plus γ-PGA; Group 3 (PGA + Gd^3+^) with half-strength modified HS plus γ-PGA and Gd^3+^ (100 μM GdCl_3_: a Ca^2+^ signaling blocker); and Group 4 (PGA + DPI) with half-strength modified HS plus γ-PGA and 100 μM DPI. The blockers were added 24 h before the addition of γ-PGA. Forty-eight hours after the addition of γ-PGA, the contents of H_2_O_2_ and Ca^2+^ in roots and leaves were determined. We also determined Ca^2+^ and H_2_O_2_ fluxes in the root tip and stem transverse sections.

H_2_O_2_ and Ca^2+^ fluxes were determined using the non-invasive micro-test technique (NMT system BIO-IM; Younger Corp., Falmouth, MA, USA)^54^. Briefly, the fresh excised root tip or stem section was fixed and immersed in a culture dish containing a base solution (BS) (0.1 mM NaCl, 0.1 mM MgSO_4_, 0.1 mM KCl, 0.1 mM CaCl_2_, and 0.3 mM MES, pH 5.8). The microelectrode of the NMT system was placed 20 μm above the detection surface and then moved between two positions, 20 and 50 μm above the detection surface. The root region used to obtain measurements was the root-hair zone, close to the elongation zone that showed the highest H_2_O_2_ and Ca^2+^ fluxes in pre-experiment scanning. In the stem, the middle of the transverse region was used for detection, where the microelectrode could sense the fluxes of the whole section.

### Experiment 6

Root protoplasts were prepared as described for Experiment 2. To determine H_2_O_2_ changes in protoplasts after γ-PGA treatment, DCFH-DA (10 μM) was added to the protoplast suspension and incubated at 37 °C for 10 min. Protoplasts were washed three times with CPW-20S and divided into two groups. Protoplasts of one group were observed immediately after the addition of γ-PGA (20 mg/L) using an LSCM. To the other group of protoplasts, we added DPI (100 μM) and then 10 min later added γ-PGA, after which the protoplast were immediately observed using an LSCM. To detect the H_2_O_2_ flux in protoplasts, the protoplast suspension was centrifuged and the pellet was re-suspended in BS containing 20% sucrose (BS-20S). NMT was then used to detect changes in H_2_O_2_ flux immediately after the addition of γ-PGA or DPI (100 μM).

### Experiment 7

Rape seedlings were divided into two groups as described in Experiment 4. The roots were harvested at 0, 0.5, 1, 3, 6, 12, 24, and 48 h post-treatment. RNA was extracted from the roots and subjected to quantitative real-time PCR (qRT-PCR) analysis of *Rboh* genes in accordance with our previously described method^18^. qRT-PCR was conducted on the ABI StepOnePlus™ system. The machine output CT (Cycle Threshold) values as results. Based on international general principles, the *actin* gene was used as a reference gene. The results were calculated using the 2^−ΔΔCT^ method. The relative transcript level of each gene at 0 h was defined as 1. Sequences of *actin* and *Rboh* genes were obtained from NCBI (http://www.ncbi.nlm.nih.gov/). The primer sequences used for amplification of the 10 *Rboh* genes are shown in Table 1.

### Statistical analysis

All experiments were performed in biological and technical triplicates. Data are expressed as the mean ± standard deviation. Statistical analysis was conducted by one-way ANOVA followed by Duncan’s test (*p* < 0.05) using SPSS 17.0 software.

## Additional Information

**How to cite this article:** Lei, P. *et al*. The microbe-secreted isopeptide poly-γ-glutamic acid induces stress tolerance in *Brassica napus* L. seedlings by activating crosstalk between H_2_O_2_ and Ca^2+^. *Sci. Rep.* **7**, 41618; doi: 10.1038/srep41618 (2017).

**Publisher's note:** Springer Nature remains neutral with regard to jurisdictional claims in published maps and institutional affiliations.

## Figures and Tables

**Table 1:** Primer sequences used for qRT-PCR.

Gene (Accession No.)	Primer Sequences
*actin* (AF111812)	L1-5′ AAGAGCTGGAGACGGCTAAG 3′
	R1-5′ GTACTTCAGGGCAACGGAAT 3′
*Rboh A* (XM_013844050)	L1-5′ CCATCTCCGTGTACAACTCG 3′
	R1-5′CAGCACCGAGATCTTCTTCA 3′
*Rboh B* (XM_013860736)	L1-5′ GACGAGTTCCTCAGCATTCA 3′
	R1-5′ CTGTGAAGAGCCCACTTTGA 3′
*Rboh C* (XM_013823796)	L1-5′ CAAAGAACAAGCCGAACTCA 3′
	R1-5′ ACCAGTCGAAAGAGCCTTGT 3′
*Rboh D* (XM_013788801)	L1-5′ TGTAGAGCCGTCTCTCCCTT 3′
	R1-5′ ACGGTCCTGAGCTTACGAGT 3′
*Rboh E* (XM_013804741)	L1-5′ CTTGTGCCATAGTGATTGGG 3′
	R1-5′ CCATTGAAGGGAGAAGCAAT 3′
*Rboh F* (XM_013825233)	L1-5′ AGAACGTTGAAGGGTGGAAC 3′
	R1-5′ AGCTCCAATGCAAACTCCTT 3′
*Rboh G* (XM_013850524)	L1-5′ TGCACCAGCACAAGACTACA 3′
	R1-5′ TCTCCATTTGGTGGAGTTGA 3′
*Rboh H* (XM_013791424)	L1-5′ TTCAACATGCCAAGAATGGT 3′
	R1-5′ ATGCTTCGTGTTTGCTTGAC 3′
*Rboh I* (XM_013838445)	L1-5′ CATGAGCGAAATTGCTGACT 3′
	R1-5′ ACGTTTGGATCATGGTGAGA 3′
*Rboh J* (XM_013791445)	L1-5′ CATTGTTTCTGAGAGCCGAA 3′
	R1-5′ GAAGTTTCGTGCTTGCTTGA 3′
